# Accuracy of pulse oximetry to determine severe hypoxemia in COPD patients: a cross-sectional study

**DOI:** 10.1016/j.clinsp.2026.101227

**Published:** 2026-11-05

**Authors:** Luis Maurício Batalin Júnior, Jonathas William de Morais, Maércio Souza Cícero dos Santos, Luís Fernando Pereira Brizola, Matheus Augusto Callegari, Danilo Augusto Ferrari Dias, Flávio Vinicius Fagundes Xavier, Estefânia Aparecida Thomé Franco, Jessica Geronutti, Lucas André Guiral Oliveira, Fabio Kendi Yamauchi, Irma de Godoy, Suzana Erico Tanni, Robson Aparecido Prudente

**Affiliations:** a Faculdade de Medicina de Botucatu (FMB), Universidade Estadual Paulista (UNESP), Botucatu Campus, Botucatu, SP, Brazil; b Hospital das Clínicas da Faculdade de Medicina de Botucatu (HCFMB), Universidade Estadual Paulista (UNESP), Botucatu Campus, Botucatu, SP, Brazil

**Keywords:** COPD, Hypoxemia, Pulse oximetry, Arterial blood gas, Long-term oxygen therapy

## Abstract

**Background:**

In moderate hypoxemia, Pulse Oximetry (SpO_2_) may indicate values above 90%, potentially underestimating severity in patients with COPD. This leads to the question of whether a similar discrepancy occurs in cases of severe hypoxemia. This study aimed to evaluate the diagnostic accuracy of SpO_2_ for identifying severe resting hypoxemia defined by PaO_2_ ≤55 mmHg and to determine a clinically applicable SpO_2_ cutoff for screening patients with COPD undergoing evaluation for LTOT.

**Methods:**

Data were collected from medical records of patients attending a Long-Term Oxygen Therapy (LTOT) outpatient clinic. Severe hypoxemia was defined as PaO_2_ ≤55 mmHg. Agreement between SpO_2_ and co-oximetry (SaO_2_) was assessed following Bland and Altman recommendations. Receiver Operating Characteristic (ROC) curve analysis was performed using a PaO_2_ ≤ 55 mmHg cutoff to evaluate the accuracy of SpO_2_.

**Results:**

Between January 2022 and December 2025, 176 patients were included (65% female, mean age: 71-years [SD = 9] and PaO_2_: 57.4 mmHg [SD = 10.8]). Bland-Altman analysis showed a mean bias of -0.43% (95% CI: -0.76 to -0.10) between SaO_2_ and SpO_2_, with 95% limits of agreement ranging from -4.77% to 3.90%. ROC curve analysis demonstrated excellent diagnostic performance of SpO_2_ for detecting severe hypoxemia, with an area under the curve of 0.939. The optimal statistical cutoff was 89.5%, corresponding to a clinically applicable threshold of SpO₂ ≤ 89%, and yielded a sensitivity of 88.5%, specificity of 87.8%, positive predictive value of 85.2%, and negative predictive value of 90.5%.

**Conclusions:**

Pulse oximetry demonstrated excellent diagnostic accuracy for detecting severe hypoxemia in COPD outpatients. An SpO_2_ cutoff of ≤89% provided optimal sensitivity and specificity under standardized conditions.

## Highlights

• Pulse oximetry accurately identified severe hypoxemia in COPD.
• SpO_2_ ≤ 89% showed high sensitivity for LTOT screening.
• ROC analysis showed excellent diagnostic performance of SpO_2_.
• Bland-Altman analysis showed good agreement between SpO2 and SaO_2_.
• Standardized assessment improved SpO_2_ accuracy in stable COPD.

## Introduction

Long-Term Oxygen Therapy (LTOT) is one of the few interventions proven to improve survival in patients with Chronic Obstructive Pulmonary Disease (COPD) and severe resting hypoxemia. Current international guidelines recommend LTOT initiation when the partial pressure of oxygen in arterial blood (PaO_2_) is ≤ 55 mm Hg, or ≤ 59 mm Hg in the presence of signs of tissue hypoxia such as pulmonary hypertension, right heart failure, or secondary polycythemia.^1^^,^^2^

Arterial Blood Gas (ABG) analysis remains the gold standard for confirming these criteria; however, pulse Oximetry (SpO_2_) is widely used as a non-invasive screening tool due to its practicality, accessibility, and ability to provide immediate results. While previous studies have suggested that SpO_2_ may underestimate the severity of moderate hypoxemia, it is less clear how reliable it is for detecting severe hypoxemia, which is the critical threshold for LTOT eligibility.^3^^,^^4^ False-negative results in this context may delay treatment initiation, with potential adverse impacts on morbidity and mortality. Conversely, false-positive results may lead to unnecessary ABG testing, increasing healthcare costs and patient burden. Given the clinical and economic implications of accurate LTOT assessment, it is essential to establish SpO_2_ thresholds that maximize both sensitivity and specificity for detecting severe hypoxemia in stable COPD outpatients. This study aimed to evaluate the diagnostic accuracy of SpO_2_ for identifying severe resting hypoxemia, defined by PaO_2_ ≤55 mmHg, and to determine a clinically applicable SpO_2_ cutoff for screening patients with COPD undergoing evaluation for LTOT.

## Methods

### Study design and population

This cross-sectional observational study was based on data extracted from medical records of patients attending the LTOT outpatient clinic at the Clinical Hospital of the Faculty of Medicine of Botucatu – HCFMB/UNESP, in the city of Botucatu, SP, Brazil, from January 2022 to December 2025.

This study involving human participants was reviewed and approved by the Research Ethics Committee of Botucatu Medical School (approval ID: 60430116.2.0000.5411). All procedures were conducted in accordance with the ethical standards of the institutional and/or national research committee and with the 1964 Declaration of Helsinki and its later amendments or comparable ethical standards. Written informed consent was obtained from all participants prior to their inclusion in the study.

Patients were instructed to be seated and to breathe room air for 30-minutes prior to oxygen saturation (SpO_2_) assessment and ABG sampling. SpO_2_ measurements were obtained using the DX 2022 Patient Monitor (Dixtal Biomédica [now Philips], São Paulo, SP, Brazil), with readings recorded after 30 seconds and confirmation of a stable waveform on the monitor. Arterial blood samples were consistently collected and immediately sent for analysis using a GEM Premier 5000 blood gas analyzer (Instrumentation Laboratory, Bedford, MA, USA). For the agreement analysis, SaO₂ values obtained by co-oximetry were used; these values corresponded to functional oxygen saturation. Severe resting hypoxemia was defined as PaO_2_ ≤55 mm/Hg. The barometric pressure in Botucatu-SP is approximately 934 hPa. Additional data collected included demographic characteristics, full ABG analysis, smoking status, and the Charlson Comorbidity Index (CCI). The CCI is a validated prognostic tool that assigns weighted scores to specific comorbidities according to their impact on long-term mortality.^5^

The inclusion criteria were patients aged ≥ 40-years with a confirmed diagnosis of COPD, defined by the presence of dyspnea, chronic cough or sputum production, and/or a history of exposure to risk factors for the disease, plus a post-bronchodilator FEV₁/FVC < 0.7 on spirometry, who had undergone at least one evaluation in the LTOT clinic for program follow-up. Exclusion criteria included non-COPD, pulmonary hypertension or severe cardiovascular disease, absence of Arterial Blood Gas (ABG) analysis, and other exclusion criteria, including incomplete medical records, recent clinical instability or COPD exacerbation, inability to perform room-air assessment, missing SpO_2_ data, duplicate evaluations, and technical issues during measurement procedures. Data completeness for each study variable is presented in Supplementary Table S1. Missing data were treated as missing, and no imputation methods were applied.

### Data analysis

The minimum required sample size was calculated according to Buderer’s method for diagnostic accuracy studies.^6^^,^^7^ The primary outcome was the diagnostic accuracy of SpO_2_ for identifying severe resting hypoxemia, defined as PaO_2_ ≤ 55 mmHg. The calculation was based on the observed prevalence of severe hypoxemia in the study population (47%) and previously published estimates of pulse oximetry performance (sensitivity and specificity of approximately 92%).^3^ Assuming a 95% confidence level and a maximum acceptable margin of error of 10%, a minimum of 60 participants was required.

Descriptive statistics were applied to delineate the characteristics of all participants. Normally distributed variables were presented as mean and standard deviation, while non-normally distributed variables were reported as median and 25%–75% percentiles. Agreement between SpO_2_ and SaO_2_ was assessed following Bland and Altman’s recommendations.^8^ The mean of both methods was plotted against their difference. Bias (systematic error) was determined by the mean difference between SpO_2_ and SaO_2_, while precision (random error), was assessed by the Standard Deviation (SD) of this difference. The 95% limits of agreement were computed as the mean difference ± 1.96 SD. A positive bias indicates that pulse oximetry overestimates SaO_2_, while a negative bias indicates underestimation. Sensitivity, specificity, false negatives, and false positives were calculated using SpO_2_ and PaO_2_ values. Receiver Operating Characteristic (ROC) curve analysis was performed using severe resting hypoxemia (PaO_2_ ≤55 mmHg) as the reference standard to evaluate the diagnostic accuracy of SpO_2_. The optimal SpO_2_ cutoff was determined using Youden’s index. A 5% significance level was applied to all tests. The statistical analyses were performed using Jamovi version 2.3 (The Jamovi project, Sydney, Australia) and SPSS version 20.0 (IBM Corp., Armonk, NY).

## Results

Between January 2022 and December 2025, 516 patients were evaluated for LTOT, of whom 176 were included in the final analysis (Fig. 1). Of these, 78 (44.3%) had PaO_2_ ≤55 mmHg. The mean age was 71-years (SD = 9), and 65% were female. Baseline characteristics are presented in Table 1.

**Fig. 1:**
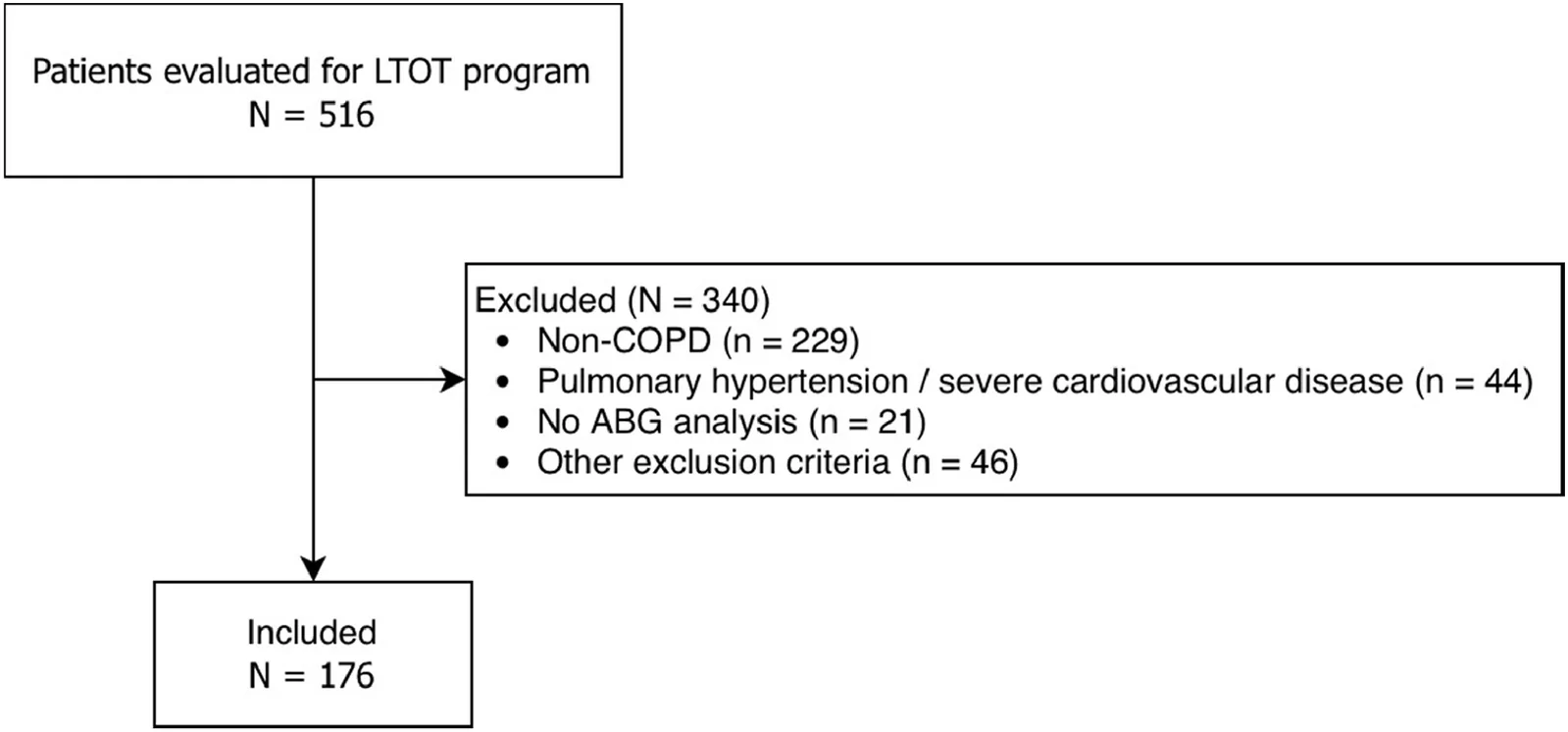
Flowchart of participant inclusion and assessment. Note: Other exclusion criteria included incomplete medical records, recent clinical instability or COPD exacerbation, inability to perform room-air assessment, missing SpO_2_ data, duplicate evaluations, and technical issues during measurement procedures.

**Table 1:** Baseline characteristics of the study population.

Characteristics	
Female sex, n (%)	115 (65)
Age, years, mean (SD)	71 (9)
Race / Ethnicity	
White, n (%)	157 (89)
Black, n (%)	8 (5)
Mixed-race, n (%)	9 (5)
Asian, n (%)	2 (1)
BMI, kg/m^2^, median (IQR)	26 (21–30)
Smoking history	
Former smokers, n (%)	144 (82)
Active smokers, n (%)	32 (18)
Charlson Comorbidity Index, median (IQR)	3 (2–5)
mMRC, median (IQR)	2 (2–3)
Post-bronchodilator Spirometry	
FEV1, %, median (IQR)	38 (31–50)
FVC, %, median (IQR)	60 (53–71)
FEV1/FVC, ratio, median (IQR)	50 (41–57)
Hematocrit, (%) median (IQR)	44 (40–47)
SpO_2_, median (IQR)	90 (86–94)
Arterial Blood Gas analysis	
pH, median (IQR)	7.40 (7.38–7.43)
PaO_2_, mmHg, mean (SD)	57.5 (10.8)
PaCO_2_, mmHg, median (IQR)	42.1 (37.8–47.2)
HCO_3_^-^, mmol/L, median (IQR)	26.3 (24.1–29.0)
BE, mmol/L, median (IQR)	1.3 (-0.6–3.7)
SaO_2_, %, median (IQR)	89.3 (85.5–92.5)

Data are presented as mean ± Standard Deviation (SD) or median [Interquartile Range (IQR)]. BMI, Body Mass Index; SD, Standard Deviation; CCI, Charlson Comorbidity Index; IQR, Interquartile Range (25^th^–75^th^ percentile); mMRC, modified Medical Research Council dyspnea scale; FEV_1_, Forced Expiratory Volume in 1 second; FVC, Forced Vital Capacity; PaO_2_, Arterial Partial Pressure of Oxygen; PaCO_2_, Arterial Partial Pressure of Carbon Dioxide; HCO₃⁻, Bicarbonate; BE, Base Excess; SaO_2_, Arterial Oxygen Saturation.

For the agreement between SaO_2_ and SpO_2_, the mean bias was -0.43%, with a 95% Confidence Interval (95% CI) ranging from -0.76 to -0.10. The 95% limits of agreement ranged from -4.77 to 3.90 (Fig. 2).

**Fig. 2:**
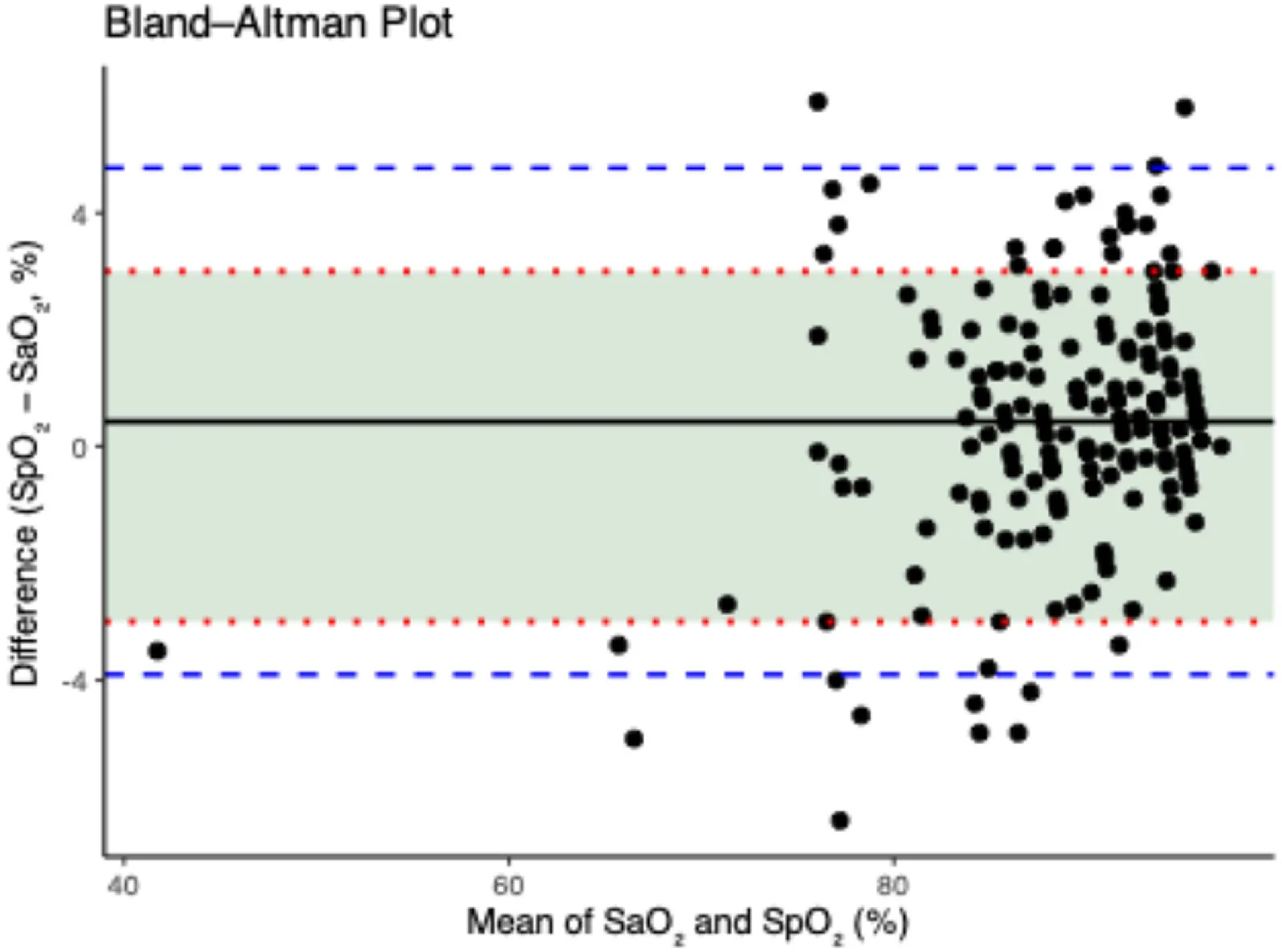
Bland-Altman plot showing the agreement between peripheral oxygen saturation measured by pulse oximetry (SpO_2_) and arterial oxygen saturation (SaO_2_). The x-axis represents the mean of the SpO_2_ and SaO_2_ measurements, and the y-axis represents the difference between the two methods (SpO_2_ – SaO_2_). The solid line represents the mean bias, and the dashed lines represent the 95% limits of agreement (mean difference ±1.96 standard deviations). The dotted lines indicate differences of ±3%, and the shaded area represents the range between these thresholds.

The Area Under the ROC Curve (AUC) for severe hypoxemia was 0.939. The optimal statistical cutoff identified using Youden's index was 89.5%, yielding a sensitivity of 88.5% and specificity of 87.8%. Because SpO_2_ is recorded as whole-number values, the ROC-derived cutoff of 89.5% represents the threshold separating values ≤ 89% from those ≥ 90%; therefore, SpO_2_ ≤ 89% was considered the clinically applicable threshold. At this threshold, the Positive Predictive Value (PPV) was 85.2%, the Negative Predictive Value (NPV) was 90.5%, and Youden's index was 0.762 (Fig. 3).

**Fig. 3:**
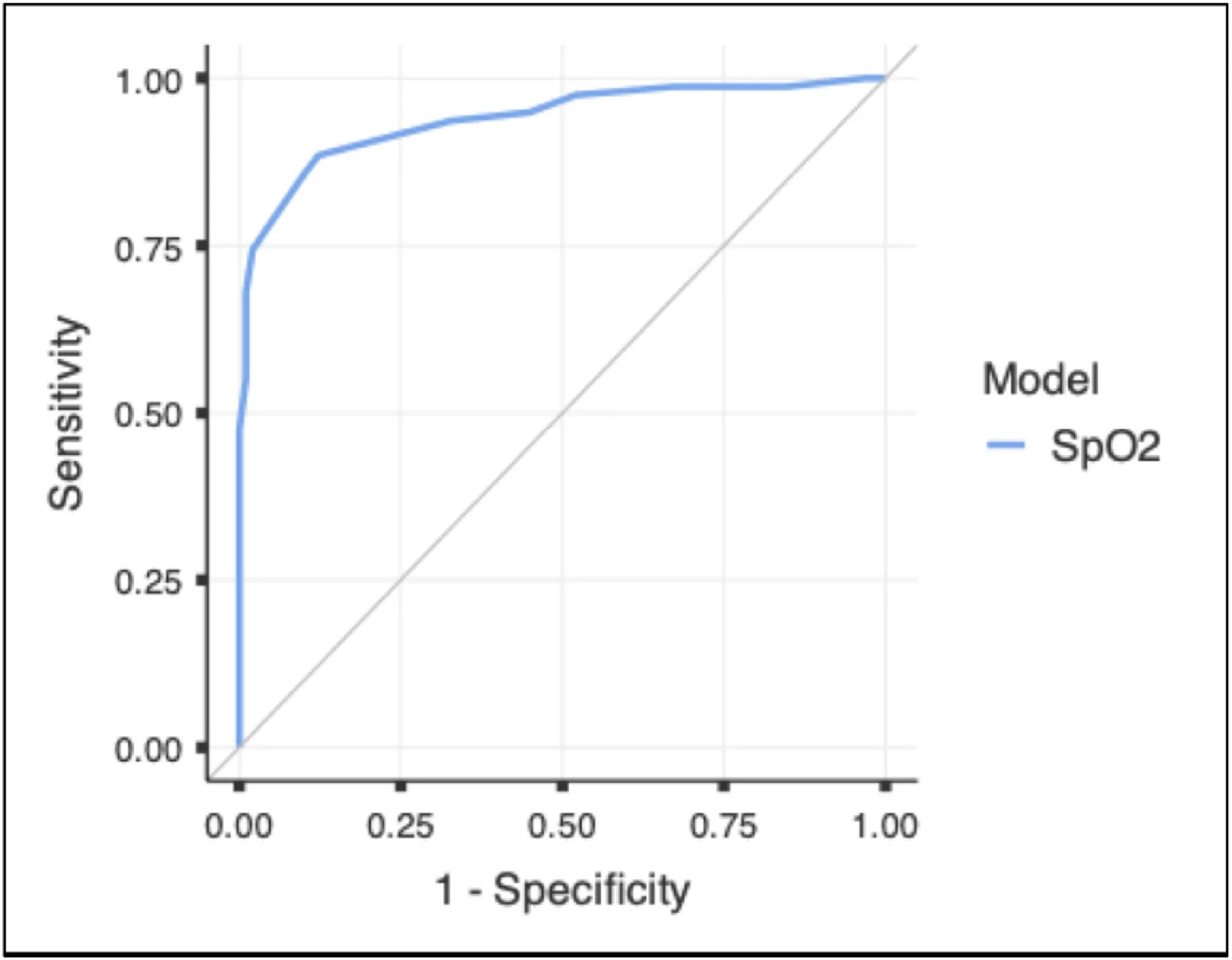
ROC Curve assessing the accuracy of SpO_2_ in detecting severe hypoxemia (PaO_2_ ≤ 55 mm/Hg).

## Discussion

The main finding of this study was that pulse oximetry demonstrated excellent diagnostic performance for identifying severe resting hypoxemia, defined by the PaO_2_ threshold used for Long-Term Oxygen Therapy (LTOT) eligibility, in stable COPD outpatients evaluated under strictly standardized conditions. ROC curve analysis showed an AUC of 0.939, and an SpO_2_ cutoff of ≤ 89% yielded high sensitivity and specificity. These findings support the use of pulse oximetry as a first-line screening tool for severe hypoxemia in stable COPD outpatients before confirmatory arterial blood gas analysis.

The agreement analysis between SpO_2_ and SaO_2_ was included as a complementary assessment of measurement performance rather than as the primary focus of the study. Overall, pulse oximetry showed good agreement with SaO_2_, with a small negative mean bias that was unlikely to be clinically relevant. Although the limits of agreement indicated some degree of variability, they were considered clinically acceptable for screening purposes. Some dispersion persisted at lower saturation levels (SpO_2_ < 80%), a range in which reduced oximeter precision is well recognized.^9^ Importantly, this variability did not substantially affect the ROC-derived cutoff or the overall diagnostic performance of SpO_2_.

The clinical relevance of our findings lies primarily in the identification of a clinically applicable SpO_2_ threshold for screening severe hypoxemia in a homogeneous population of stable COPD patients undergoing evaluation for LTOT. When measurements were obtained under strictly standardized conditions, including clinical stability and controlled assessment procedures, pulse oximetry demonstrated excellent diagnostic performance. This context-specific approach may help explain part of the variability reported in previous studies conducted in more heterogeneous populations or less controlled clinical settings.

However, the literature reports variable performance of SpO_2_ in this context, particularly with respect to sensitivity.^3^^,^^4^ Garnet et al., in a study also conducted in patients with COPD undergoing evaluation for LTOT eligibility, reported reduced sensitivity when similar cutoffs were applied, with a higher false-negative rate when pulse oximetry was used in isolation. Importantly, the same authors demonstrated a progressive improvement in sensitivity with higher SpO₂ thresholds, suggesting that diagnostic performance is strongly influenced by clinical and methodological context.^3^ Our findings suggest that, when SpO_2_ is measured in a clinically stable population under strictly standardized conditions, its diagnostic performance may be optimized.

This interpretation is supported by studies that have also demonstrated good SpO_2_ performance in controlled settings. Garcia-Gutierrez et al. reported a strong correlation between SpO_2_ and PaO_2_ in patients with COPD exacerbations, with optimal SpO_2_ values close to 90% for identifying clinically significant hypoxemia.^10^ Similarly, Razi et al. reported high sensitivity and specificity of pulse oximetry in patients with pulmonary diseases, although they observed reduced accuracy at very low saturation levels (< 80%).^11^ Notably, this extreme saturation range was not determinant for the cutoff identified in the present study.

Overall, our findings reinforce that pulse oximetry provides a reliable estimate of arterial oxygen saturation in most clinical scenarios, and that its limitations are context-dependent rather than intrinsic to the method itself. In this regard, the use of a standardized measurement protocol and the exclusion of potentially confounding respiratory conditions allowed the establishment of a more homogeneous cohort of stable COPD patients, strengthening the internal validity of the study. Patients with other respiratory diseases were excluded to avoid pathophysiological heterogeneity, as different mechanisms of hypoxemia (e.g., shunt, diffusion limitation, interstitial involvement) may influence the relationship between SpO_2_ and PaO_2_. Therefore, the identified cutoff should not be directly extrapolated to non-COPD populations. These same considerations help explain why, in less controlled clinical environments and more heterogeneous populations (common in routine practice), the diagnostic performance of SpO_2_ may be inferior to that observed in the present analysis.

Finally, physiological factors such as hypercapnia and elevated carboxyhemoglobin levels (for example, related to smoking) may reduce the accuracy of pulse oximetry, with a tendency toward overestimation of arterial oxygen saturation.^12^^,^^13^ Given that our analysis included patients with varying degrees of hypercapnia and did not distinguish those with active smoking, these factors may have contributed to residual measurement bias and should be considered when interpreting individual SpO₂ values. Thus, while pulse oximetry is an effective screening tool, arterial blood gas analysis remains essential for diagnostic confirmation in borderline cases or when clinical findings are discordant.

An additional limitation is the single-center design, which may limit the generalizability of the findings to other clinical settings. Furthermore, although pulse oximetry was assessed under standardized conditions using a single device, the findings should be interpreted within the context of the measurement protocol used in this study. Other factors that may affect SpO_2_ accuracy, such as skin pigmentation and peripheral perfusion status, were not systematically assessed and may have contributed to variability in measurements.^14^^,^^15^

## Conclusion

Pulse oximetry demonstrated good overall agreement with co-oximetry and excellent diagnostic performance for identifying severe hypoxemia in stable COPD outpatients. In this setting, an SpO_2_ cutoff of ≤89% showed high sensitivity and specificity for detecting severe resting hypoxemia, fulfilling the objective of determining a clinically useful threshold with good diagnostic performance. These findings support the use of pulse oximetry as a first-line screening tool under the standardized measurement conditions evaluated in this study.

## Declaration of AI use

Artificial intelligence-assisted language editing tools were used to support the translation and language revision of the manuscript. All scientific content, data interpretation, and final revisions were performed and approved by the authors.

## Authors’ contributions

LMBJ, JWM, IG, SET, and RAP designed the research; LMBJ, JWM, MSCS, LFPB, MAC, DAFD, FVFX, EATF, JG, LAGO, and FKY conducted the research; RAP and SET analyzed the data; LMBJ and JWM wrote the original draft; and LMBJ, JWM, SET, and RAP performed the review and editing. All authors read and approved the final manuscript.

## Funding

São Paulo Research Foundation (FAPESP), grant number 2024/07508-4.

## Data availability statement

The data that support the findings of this study are available from the corresponding author upon reasonable request.

## Conflicts of interest

The authors declare that the research was conducted in the absence of any commercial or financial relationships that could be construed as a potential conflict of interest.
