# Supplementary material for: Accuracy of pulse oximetry to determine severe hypoxemia in COPD patients: a cross-sectional study
Source: Clinics (Sao Paulo). 2026 Nov 5;81:101227. doi: 10.1016/j.clinsp.2026.101227 (PMC13769088; doi:10.1016/j.clinsp.2026.101227)
Supplement: Supplementary file 1 [file mmc1.docx]

**CLINICS-D-26-01453_ Supplementary Material**

**Supplementary Table S1** Completeness of data for study variables.

| **Characteristics** | **Available, n (%)** | **Missing, n (%)** |
| --- | --- | --- |
| Sex | 176 (100.0) | 0 (0.0) |
| Age | 176 (100.0) | 0 (0.0) |
| Race / Ethnicity | 176 (100.0) | 0 (0.0) |
| BMI | 174 (98.9) | 2 (1.1) |
| Smoking history | 176 (100.0) | 0 (0.0) |
| Charlson Comorbidity Index | 170 (96.6) | 6 (3.4) |
| mMRC | 174 (98.9) | 2 (1.1) |
| Post-bronchodilator Spirometry | 163 (92.6) | 13 (7.4) |
| Hematocrit, % | 167 (94.9) | 9 (5.1) |
| SpO_2_ | 176 (100.0) | 0 (0.0) |
| Arterial Blood Gas analysis |  |  |
| pH | 176 (100.0) | 0 (0.0) |
| PaO_2_ | 176 (100.0) | 0 (0.0) |
| PaCO_2_ | 176 (100.0) | 0 (0.0) |
| HCO_3_^-^ | 176 (100.0) | 0 (0.0) |
| BE | 156 (88.6) | 20 (11.4) |
| SaO_2_ | 176 (100.0) | 0 (0.0) |

BMI, Body Mass Index; SD, Standard Deviation; CCI, Charlson Comorbidity Index; IQR, Interquartile Range (25^th^–75^th^ percentile); mMRC, modified Medical Research Council dyspnea scale; FEV_1_, Forced Expiratory Volume in 1 second; FVC, Forced Vital Capacity; PaO_2_, Arterial Partial Pressure of Oxygen; PaCO_2_, Arterial Partial Pressure of Carbon dioxide; HCO₃⁻, Bicarbonate; BE, Base Excess; SaO_2_, Arterial Oxygen Saturation.
